# PARP-1 cleavage fragments: signatures of cell-death proteases in neurodegeneration

**DOI:** 10.1186/1478-811X-8-31

**Published:** 2010-12-22

**Authors:** Ganta Vijay Chaitanya, Jonathan S Alexander, Phanithi Prakash Babu

**Affiliations:** 1 Department of Biotechnology, School of Life Sciences, University of Hyderabad, Hyderabad, India; 2 Department of Molecular and Cellular Physiology, Louisiana State University Health Sciences Center-Shreveport, Louisiana-USA

## Abstract

The normal function of poly (ADP-ribose) polymerase-1 (PARP-1) is the routine repair of DNA damage by adding poly (ADP ribose) polymers in response to a variety of cellular stresses. Recently, it has become widely appreciated that PARP-1 also participates in diverse physiological and pathological functions from cell survival to several forms of cell death and has been implicated in gene transcription, immune responses, inflammation, learning, memory, synaptic functions, angiogenesis and aging. In the CNS, PARP inhibition attenuates injury in pathologies like cerebral ischemia, trauma and excitotoxicity demonstrating a central role of PARP-1 in these pathologies. PARP-1 is also a preferred substrate for several 'suicidal' proteases and the proteolytic action of suicidal proteases (caspases, calpains, cathepsins, granzymes and matrix metalloproteinases (MMPs)) on PARP-1 produces several specific proteolytic cleavage fragments with different molecular weights. These PARP-1 signature fragments are recognized biomarkers for specific patterns of protease activity in unique cell death programs. This review focuses on specific suicidal proteases active towards PARP-1 to generate signature PARP-1 fragments that can identify key proteases and particular forms of cell death involved in pathophysiology. The roles played by some of the PARP-1 fragments and their associated binding partners in the control of different forms of cell death are also discussed.

## Introduction

PARP-1 is a nuclear protein with a wide range of physiological as well as pathological functions. Initially identified as an enzyme that performs central roles in the repair of damaged DNA, PARP-1 participates in initiating base excision repair (BER) (PARP-1^-/- ^cells have impaired BER activity) [1,2], nucleotide excision repair, single strand base repair mediated by DNA ligase III, XRCC1, poly nucleotide kinase, proliferating cell nuclear antigen and flap endonuclease-1, and contributes to double strand base (DSB) repair in an alternate non-homologous end joining pathway with DNA ligase III [3-6]. Interestingly, over-expression of PARP-1 or DNA binding domain of PARP-1 (lacking catalytic domain) decreased DSB repair, indicating that its enzymatic activity is not essential in all repair processes [7]. Many additional functions of PARP-1 have now been demonstrated in biochemical and molecular signaling [8]. Apart from its role in repairing DNA damage, PARP-1 also plays important roles in transcription, cardiac remodeling, vasoconstriction, regulation of astrocyte and microglial function, long term memory and aging [9-17]. Progressive DNA damage and decreased PARP-1 activity in aging neurons eventually leads to programmed neuronal death and loss of memory consolidation. PARP-1's role in neuronal BER indicates that it may influence age-related memory deficits and dementia. Further, over-expression of PARP-1 and telomeric repeat binding factor-1 were also associated with age dependent telomere shortening in 'Duchenne muscular dystrophy' [18]. PARP-1 influences ~3.5% of the total transcriptome of embryonic liver and stem cells and regulates ~60-70% of genes controlling cell metabolism, cell cycle and transcription. Gene expression is dysregulated in PARP-1 deficient fibroblasts and PARP-1 deficient mice are more susceptible to skin diseases [19-21] reflecting the role of PARP-1 against UV-induced DNA damage. PARP-1 also interacts with, and modulates the function of several transcription factors including NF-κB, NFAT, E2F-1, and ELK-1 [22-28]. PARP-1 is also involved in modulating endothelial cell adhesion molecule expression (e.g. during atherogenesis) via its binding partner NF-κB [16,29,30]. PARP-1, 2 and 3 can activate CNS immune responses by promoting astrocyte production of inflammatory cytokines like TNF-α, IL-1β, nitric oxide and the chemokine CCL2 after challenge with *Staphylococcus aureus*, a common CNS infectious agent [14].

The PARP family consists of 17 members which have different structures and diverse functions in cells [31]. PARP-1, the canonical representative of this superfamily has become the major focus of research due to its multifaceted roles in many cellular activities. This review focuses on the interactions between PARP-1 and suicidal proteases like caspase, calpain, granzyme, and MMPs that lead to the formation of PARP-1 proteolytic signature fragments associated with particular pathological conditions.

PARP-1 is an abundant nuclear enzyme with approximately 1-2 million copies in the cell which account for ~85% of total cellular PARP activity [31-34]. Post-translation modification involving poly (ADP-ribosyl)ation plays a central role in cellular homeostasis [35]. Protein modifications involving phosphorylation, acetylation, methylation and poly (ADP ribosyl)ation are vital cellular processes that are required for cell signaling, survival and functioning [34,36-39]. This form of post translational modification is mainly mediated by PARP-1 which catalyzes the formation of chains approximately 200 units long linear or branched poly (ADP ribose) units from donor NAD^+ ^molecules frequently linked by esterification to glutamate, and less commonly to aspartate or lysine resides on target molecules [34,40]. Poly (ADP-ribosyl)ation is therefore an important mechanism for maintaining genome integrity, replication, transcription, protein degradation, differentiation and in the repair process following DNA damage [13,34,41-43].

PARP-1 has several important domains: a 54-kD catalytic domain (CD) at the carboxyl terminus that polymerizes linear or branched poly-ADP ribose units (from NAD^+^) on target proteins, a 46-kD DNA binding domain (DBD) containing 2 zinc finger motifs (at the NH_2 _terminus), and 22-kD auto-modification domain (AMD) that functions as a target for direct covalent auto-modification in its central region [34]. For example, high affinity binding of PARP-1 to specific DNA motifs like double-strand breaks, cruciforms, cross-overs and nucleosomes require the DBD for active modification [44-46]. While the 2 zinc finger motifs at the N-terminus facilitate tight binding of PARP-1 to DNA and promotes the activation of the catalytic domain at the C-terminus, a 3^rd ^zinc finger motif located between 2^nd ^zinc finger motif and AMD also plays an important role in the inter-domain interactions and is vital for PARP-1 enzymatic action [47]. AMD contains a BRCT fold (a motif also found in many DNA repair proteins) that is involved in protein-protein interactions which promotes the recruitment of DNA repair enzymes to the site of DNA damage [48,49]. These PARP-1 domains play different roles in various pathological cell death processes mediated by PARP-1 cleavage by suicide proteases. These suicidal proteases (caspases, calpains, cathepsins, granzymes and MMPs) cleave PARP-1, creating PARP-1 fragments with diverse, exposed structural domains mediating specific forms of cell death. The present review focuses on the different actions of these proteases towards PARP-1, the production of a variety of different PARP-1 signature fragments, and the specific patterns of cell death linked with particular PARP-1 fragments.

### PARP-1 and Caspases

Apoptosis, the process of programmed cell death is essential for proper homeostatic maintenance and survival in multi-cellular organisms. Physiological apoptosis controls cell numbers, tissue and organ morphology and patterning, and removes injured or mutated cells [50,51]. Dysregulated apoptosis results in elevated or decreased cell death often leading to neurodegenerative disorders, cancer and other hyper-proliferative disorders [52,53]. One of the most common signaling cascades involved in apoptosis is the activation of a highly specialized family of cysteinyl-aspartate proteases (caspases) which are usually present as inactive zymogen forms. Once activated, caspases initiate cell death by cleaving and activating effector caspases which drive the process of apoptosis [54]. Interestingly, recent reports have shown the involvement of caspases not only in apoptosis, but also in forms of cell proliferation in a *Drosophila *model. One of the 2 distinct forms of 'apoptosis induced compensatory proliferation' (AICP) depends on initiator caspase Dronc (initiator caspase in Drosophila, caspase-9-like); the other is dependent on execution caspase DrICE and Dcp-1 (effector caspases in Drosophila; caspase-3-like) [55-58]. Moreover, apart from their primary function in executing apoptosis, non-apoptotic functions of caspases include hematopoiesis (erythropoiesis, monocyte, lymphocyte differentiation, platelet maturation) [59] and regulation of neuronal synaptic plasticity in long-term potentiation [60,61]. Caspase mediated apoptotic cell death is accomplished through the cleavage of several key proteins required for cellular functioning and survival [62]. PARP-1 is one of several known cellular substrates of caspases. Cleavage of PARP-1 by caspases is considered to be a hallmark of apoptosis [63,64]. Almost all caspases including caspase-1, are known to modify PARP-1 *in vitro *[65]. Lazenbik et al., have observed protease activity resembling interleukin converting enzyme (prICE: caspase-3) which cleaves PARP-1 after aspartate (glutamate-valine-*aspartate*-glycine), a substrate specificity identical to one of ICE cleavage sites *in vitro *to yield an 85-kD PARP-1 fragment [66]. Cleavage of PARP-1 by caspase-3 has been implicated in several neurological diseases e.g. cerebral ischemia, Alzheimer's disease, multiple sclerosis, Parkinson's disease, traumatic brain injury, NMDA-mediated excitotoxicity and brain tumors, especially gliomas [67-74]. Besides caspase-3, caspase-7 also cleaves PARP-1 *in vivo*. The cleavage of PARP-1 by these caspases results in the formation of 2 specific fragments: an 89-kD catalytic fragment and a 24-kD DBD [65,66]. The 89-kD fragment containing AMD and the catalytic domain of the enzyme has a greatly reduced DNA binding capacity and is liberated from the nucleus into the cytosol [75]. The 24-kD cleaved fragment with 2 zinc-finger motifs is retained in the nucleus, irreversibly binding to nicked DNA where it acts as a trans-dominant inhibitor of active PARP-1. Importantly, irreversible binding of the 24-kD PARP-1 fragment to DNA strand breaks inhibits DNA repair enzymes (including PARP-1) and attenuates DNA repair (also conserving cellular ATP pools) [76-78]. Poly (ADP-ribosyl)ation of PARP-1 following DNA breaks changes PARP-1 targeting from caspase-3 from caspase-7 in human promyelocytic leukemia cells (HL-60) treated with etoposide phosphate (VP-16). However, increased caspase-3 levels (and activity) might lead to PARP-1 cleavage irrespective of its auto-modification (by poly (ADP-ribosyl)ation) [79]. In a similar context, Margolin et al., using the 46-kD DBD of PARP-1 (lack which auto-modification sites) reported an increased affinity of caspase-3 for PARP-1, indicating a prominent role for PARP-1 automodification sites in caspase-3 mediated proteolytic cleavage [65].

**Figure 1:**
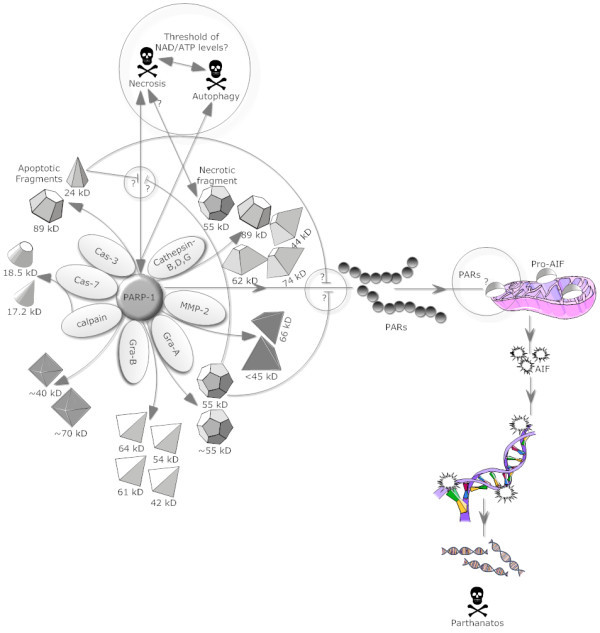
**PARP-1 interactions with cell death proteases**. PARP-1 cleavage by various suicidal proteases like caspases, calpain, cathepsins and granzymes liberates fragments with specific molecular weights and are shown in this schematic representation. Importantly, 21-kD and 55-kD PARP-1 fragments generated by caspase 3/7 and Gra-A respectively function as an inhibitor of PARP-1 activity and might also play important roles in reducing necrosis and/or parthanatos. Further, PARP-1 and PARP-1 fragment's involvement in various forms of cell death e.g. autophagy, necrosis and parthanatos are also indicated.

Under basal conditions, the primary function of PARP-1 is to detect and repair DNA damage. However, cells with severely damaged DNA have amplified PARP-1 activity resulting in high NAD^+ ^consumption (depleting ATP pools). If unchecked, this activity inevitably leads to passive necrotic cell death (resulting from prolonged ATP depletion) [80,81]. This process is blocked by rapid cleavage and inactivation of PARP-1 by the action of caspases [81,82]. However, insults which initiate necrosis cause PARP-1 overactivation that proceeds unchecked due to inadequate caspase activation [82-84], lower PARP-1 cleavage and less PARP-1 24-kD fragment formation. It is therefore possible that exogenous addition of PARP-1 24-kD fragments could attenuate PARP-1 overactivation possibly blocking cell death. Exogenous administration of 24-kD PARP-1 fragments might attenuate PARP-1 overactivation and divert necrosis towards apoptotic cell death. Taken together, these findings suggest that the PARP-1 24-kD fragment can also serve as a powerful therapy for CNS disorders like cerebral ischemia where necrotic cell death predominates within the infarct core. PARP-1 cleavage by caspases and the resulting specific fragmentation patterns are indicated in figure 1 and Additional file 1: Table-1.

### PARP-1 and Calpains

Calpains are a family of 14 Ca^2+^-activated, non-lysosomal cysteinyl proteases active at neutral pH [85]. Of all the calpain isoforms, m-calpain and μ-calpain (activated at μM-mM Ca^2+ ^concentrations) are the best understood. Alterations in intracellular calcium levels are known to initiate calpain activation often exacerbating pathological conditions [86]. For example, pathologic calpain activation aggravates neurodegenerative disorders like cerebral ischemia, neonatal hypoxia, cerebral malaria, Alzheimer's disease, Parkinson's disease, Huntington's disease, multiple sclerosis, and also contributes to injury in brain tumors, such as gliomas [68-70,87-92]. Translocation of calpains to the plasma membrane is required for optimal calpain activation. However in neurons, NMDA-mediated excitotoxicity involves calpain activation without a requirement for membrane translocation [93,94]. Calpains also play prominent roles in normal physiology e.g. neuron synaptic signaling, myoblast differentiation, cell migration, embryonic development (where m-calpain deficiency is embryonic lethal) and angiogenesis [95-98]. VEGF mediated angiogenesis involves increased Ca^2+ ^uptake in cells to activate m-calpain and VEGF induced retinal angiogenesis *in vitro *could be reversed by the calpain inhibitor SNJ-1945 [99,100]. Interestingly, PARP-1 inhibition also been shown to decrease VEGF induced angiogenesis [101]. Hence, even though calpain mediated PARP-1 proteolysis can decrease angiogenesis; calpains might still promote angiogenesis by augmenting VEGF expression [100] indicating that a bimodal activation pattern of calpain activation proportional to the magnitude of calpain mediated PARP-1 proteolysis is possible. More studies are still required to address this issue and understanding the roles played by PARP-1, calpains and VEGFs in post-ischemic cerebral angiogenesis is an area of drug development, particularly for stroke therapy.

Calcium, apart from activating calpains, also promotes PARP-1 hyper-activation through production of ROS and peroxynitrite [102]. The anti-tumor drug β-lapachone induces PARP-1 dependent cell death independent of caspases via metabolic starvation. β-lapachone treated cells also exhibit increased cytosolic Ca^2+ ^(by release of endoplasmic reticulum Ca^2+ ^stores) and ROS, resulting in PARP-1 hyper-activation. PARP-1 hyper-activation by β-lapachone was blocked by BAPTA-AM (a cytosolic Ca^2+ ^chelator) indicating that Ca^2+ ^induced ROS generation leads to PARP-1 hyper-activation and cell death [103]. Conversely, cellular Ca^2+ ^levels were altered by PARP-1 in doxorubicin-induced myocardial injury. Szenczi et al., have shown that both cardiac contractile capacity and intracellular Ca^2+ ^loading was reduced in doxorubicin-treated hearts, an effect blocked by PARP-1 inhibition. These responses may be indirect through the effects of PARP-1 on the transcription of proteins involved in Ca^2+ ^handling and Ca^2+ ^pumps [104]. PARP-1 has also been shown to modulate neuronal mitochondrial Ca^2+ ^levels [105]. PARP-1 activity raises intra-mitochondrial Ca^2+ ^levels, activating μ-calpain to release mitochondrial apoptosis inducing factor (AIF) [106]. However, because activated calpains mediate proteolytic breakdown of PARP-1, the temporal association of PARP-1 and calpain in AIF nuclear translocation may be phase-dependent, more studies will be required to address this. The roles of PARP-1 in Ca^2+ ^handling and excitotoxicity point towards pleiotropic functions of PARP-1 in pathological settings.

Calpains play crucial roles in both caspase-dependent and independent forms of apoptotic cell death and are key mediators of necrotic cell death [107,108]. A variety of cellular insults can activate calpains including increased cytosolic Ca^2+^, decreased calpastatin levels, interactions with calpain activator protein, phospholipids, and caspase activation [109-112]. Several cell substrates have been identified as calpain targets resulting in proteolytic breakdown to signature fragments of specific molecular weights [69,91,113]. Appearance of distinctive cleavage ('signature') fragments is considered to represent specific forms of calpain activation. Among the various cellular substrates of calpain, PARP-1 cleavage gives rise to a 40-kD N-terminal fragment in neuroblastoma cells (SH-5YSY) challenged with maitotoxin [114]. μ-calpain isolated from calf thymus generates ~40-70-kD N-terminal PARP-1 fragments [115]. Calpain induced 40-kD fragments were recognized by both C-2-10 and N-20 antibodies (which detect PARP-1 fragments bearing the N-terminal domain/DBD). Depending on observed molecular weight and immunoreactivity it can be presumed that this PARP-1 fragment might have DBD domain, but not AMD or catalytic domains. Whether this 40-kD fragment plays any role in PARP-1 auto-modification (as shown in studies on the 24-kD PARP-1 DBD fragment) is not yet known. This fragment appears specifically when PARP-1 is digested with calpain, and μ-calpain inhibitor reduces its appearance, indicating that it is a μ-calpain-specific PARP-1 signature fragment. We don't know whether this fragment represents a byproduct formed during calpain mediated necrotic cell death or if it can actively attenuate necrosis. Based on the findings that the zinc finger motifs present in the DBD domain of PARP-1 can facilitate irreversible binding of DBD to DNA nicks and promote transdominant inhibition of activated PARP-1, the appearance of this fragment would be consistent with its ability to attenuate PARP-1 activity during calpain mediated necrosis. Calpains are known to mediate apoptotic cell death. Whether this fragment appears only during necrotic conditions or also during calpain-mediated (caspase independent) apoptosis is unknown. Future studies on the molecular biology of this fragment and its roles in specific cell death pathways will be needed to determine its role in neuronal pathologies. So far, these fragments remain most convincingly related to necrotic modes of cell death mediated by calpain.

Apart from PARP-1, caspase-7 has also been shown to be a target of μ-calpain. μ-calpain cleaves caspase-7 to active 18.5 and 17.2-kD fragments. This calpain generated caspase-7's 17.2-kD fragment is ~18 fold more active than the 20-kD caspase-3 generated fragment of caspase-7 [116]. Whether these fragments are involved in preferential PARP-1 processing over caspase-3 is not yet known. PARP-1 cleavage by calpains results in specific fragmentation patterns which are described in figure 1 and Additional file 1: Table-1.

### PARP-1 and Cathepsins

Lysosomes were initially described as participants in catabolic autophagic cell death when exhausted or damaged organelles were digested and secreted into the cytoplasm [117]. Lysosomes and lysosomal proteases also participate in apoptotic and necrotic forms of cell death [118-120]. Cathepsins belong to the family of lysosomal proteases which are active at acidic pH. These are stored in lysosomes as inactive precursors and activated by stimuli that lower cell pH, resulting in the release of their catalytically active forms which cleave multiple targets [121]. Cathepsins can initiate apoptotic cell death independent of caspases, and are known to be key proteases in necrosis and autophagy. Increased autophagic protease levels can aggravate post-ischemic neuropathologic with injury [122]. Recent reports clearly show that the biogenesis of lysosomal cathepsins is required for necrotic cell death [118]. One of the important differences between apoptotic and necrotic forms of cell death is the protein synthesis pattern. During apoptotic cell death, normal protein synthetic processes are rapidly shut down, an event which conserves ATP, which is used to accomplish apoptosis. Conversely, the persistence of protein synthesis during necrotic cell death can deplete energy reserves accelerating necrotic cell death [118-123]. Since lysosomal biogenesis is required for necrotic cell death, persistent protein synthesis might help in lysosomal-cathepsin synthesis, which eventually spills into the cytosol, cleaving PARP-1 and contributing to cell death [118,123]. However, the numerous targets and temporal specificities of cathepsins and PARP-1 contributing to cell death mechanisms require further study.

Cathepsins are a large family of lysosomal enzymes comprising 11 cysteinyl cathepsins (and aspartyl protease cathepsin-D) which are also active at neutral pH. These have a relatively short biological half-life, but acidification of the cytosol will increase their residence time. However, due to the abundance of cathepsin B, D and L, these are most often used as markers for the involvement of lysosomes in particular forms of cell death [119]. Cathepsins B, S and G are also involved in tumor invasion (glioblastoma multiforme, breast cancer bone metastasis and colorectal tumors), endothelial proliferation and in angiogenesis (via TGF-β, VEGF and MCP-1) [124,125]. It is important to note that necrosis is common in tumor tissue and is usually accompanied by PARP-1 hyper activation. Considering that PARP-1 inhibition reduces angiogenesis, it is possible that hyperactivated PARP-1 might in part drive tumor angiogenesis and metastasis. Cathepsins and TGF-β are also involved in caspase-independent PARP-1 cleavage (an ~85-kD caspase independent PARP-1 fragment is produced by TGF-β) [126]. How these mediators drive angiogenesis or can be used in diagnosis or therapy needs further study.

Moreover, TGF-β and cathepsin-G mediated angiogenesis depends on the upregulation of VEGF [124]. Hence, breakdown of PARP-1 (which would reduce the angiogenic program) might be balanced by increased VEGF production by cathepsins. It is important to identify whether the breakdown products of PARP-1 produced by calpains or cathepsins can substitute for the PARP-1 function in angiogenic programs. More importantly, the effect of transdominant inhibition of PARP-1 by DBD in modulating angiogenic programs is worth investigating. These findings indicate that angiogenesis associated with PARP-1 signaling is highly complex, tightly controlled and pathology specific. The extent of PARP-1 and PARP-1 cleavage products' involvement in modulating angiogenic programs has yet to be fully determined.

Cysteine cathepsins also share several common targets along with PARP-1. PARP-1 was initially found to produce a 50-kD fragment (necrotic fragment) during necrotic cell death. Necrotic PARP-1 fragmentation was later characterized and the appearance of these PARP-1 fragments shown to be mediated by lysosomes and lysosomal specific proteases cathepsin-B, -D and -G. During necrosis, induced by H_2_O_2_, 10% ethanol, HgCl_2_, lysosomal extracts and cathepsin-B, PARP-1 gave rise to fragments ranging from 42-89-kD [127]. Cathepsin-B and D produce active PARP-1 fragments with molecular weights of 55-kD and 42-kD, similar to the fragments obtained from the lysosomal extracts. Inactive PARP-1 fragments with molecular weights of 74 and 62-kD are also liberated by the action of these proteases. Cathepsin-G, another lysosomal protease, also produced a similar fragmentation pattern, but with lower intensity (requiring longer incubation periods vs. cathepsin-B and D). Moreover, the 89-kD PARP-1 fragment which appears during apoptotic cell death could also be produced by cathepsin-B and D, but is not generated by cathepsin-G [127]. Cathepsin specific PARP-1 fragmentation patterns are shown in figure 1 and Additional file 1: Table-1.

### PARP-1 and granzymes

The immune system maintains an internal homeostasis by recognizing and reacting to foreign particles, abnormal or infected cells to restrict their further persistence and penetration within the host. Immune processes use highly specialized and cell-specific mechanisms and molecules to accomplish this. Induction of cytotoxicity in target cells is a multifactorial process accomplished by cytotoxic T lymphocytes (CTLs) and natural killer (NK) cells that secrete toxic and lytic proteases of the serine protease family (granzymes), perforins and granulysin [128]. About 90% of cytolytic granules stored in CTLs and NK cells contain granzymes. Granzymes are subdivided into 3 categories based upon their enzymatic similarity to chymotrypsin (chymase locus), trypsin (tryptase locus) and those that cleave after unbranched hydrophilic residues particularly after methionine (Met-ase-locus). A total of 10 granzymes have been found in the mouse (granzymes A-G and K-M), 7 in rats (A, B, C, I, J, K and M) and 5 in humans (granzymes A, B, H which are specific to human, M and tryptase2/granzyme-3). Human homologs of granzymes C-G have still not been found [129].

Granzymes are secreted from cytotoxic cells upon recognizing their target cells, with several physiological or pathophysiological consequences. Physiologically, granzymes promote extracellular matrix degradation, lymphocyte migration, cytokine production and attenuate tumor cell migration[130]. Pathological granzyme actions are important in inflammatory vascular disorders (atherosclerosis, transplant vascular disease, systemic lupus erythrematosus, autoimmune vasculitis) [131-133], chronic allergic or autoimmune diseases (arthritis, chronic allergic asthma, hypersensitive pneumonitis) [134-136] and neurodegenerative disorders (spinal cord injury, cerebral ischemia, multiple sclerosis, brain tumors like gliomas) [68,70,137-139]. Of the family of granzymes, granzyme-B (Gra-B) is considered to be the most potent apoptogenic molecule, even though granzyme-A (Gra-A) and Gra-B are the most abundant proteases in lytic granules [140]. Other members of the granzyme family perform functions outside of apoptosis and independent of caspases.

Gra-A has been shown to induce caspase-independent, but morphologically indistinguishable apoptosis. Induction of Gra-A mediated apoptosis involves activation of Gra-A activated DNase to induce single stranded nicks in DNA, breakdown of oxidative repair protein 'apurinic/apyrimidinic (AP) endonuclease' (APE), KU-70 and mitochondrial complex I protein [141-144]. Apart from lamin, which is the common substrate for Gra-A, Gra-B and caspase-3, PARP-1 is also cleaved by these 3 proteases resulting in different fragmentation patterns [66,145-147]. Importantly, even though PARP-1 is a direct substrate for Gra-A it can only cleave intracellular PARP-1 in the presence of perforins [145]. This finding also underscores the necessity of perforin for Gra-A to enter the cell to execute cell death, unlike Gra-B. Gra-A activity towards PARP-1 results in its breakdown with high efficiency after Lys^498 ^leading to the formation of a C-terminal 55-kD inactive fragment and an N-terminal fragment of similar molecular weight. Cleavage of PARP-1 at Lys^498 ^residue by Gra-A results in decreased auto-modification of PARP-1, poly (ADP ribosyl)ation and DNA repair. Gra-A mediated formation of N-terminal active 55-kD PARP-1 (after Lys^498^) may cause cells to undergo caspase-independent apoptotic cell death, rather than necrotic cell death, by decreasing the efficiency of ADP ribosylation. Since Gra-A cleaves oxidative repair enzymes and increases ROS production, it will indirectly activate PARP-1; decreased poly (ADP-ribosyl)ation counteracts these deleterious effects [145]. However, whether Gra-A has any role in '*parthanatos*' (cell death mediated by poly (ADP ribose) polymers) or necrosis is a topic for future studies. Even though cathepsin-B and Gra-A produce similar 55-kD C-terminal PARP-1 fragments, they differ in recognized cleavage sites. For example, cathepsins cleave PARP-1 at leucine^525 ^residues, but Gra-A cleaves preferentially at lysine^498 ^[127,145] resulting in the formation of N-terminal fragments that attenuate PARP-1 depletion of ATP. Unlike Gra-A, cathepsin mediated PARP-1 cleavage at Leu^525 ^results in the formation of 55-kD active C-terminal and 62-kD inactive N-terminal fragments [127]. Consequently cleavage-site specificity plays a major role in the generation of fragments derived from particular domains that differentially modulate forms of cell death (apoptosis vs. necrosis).

Gra-B was the first serine protease discovered to cleave PARP-1 during induction of cell death. Gra-B can translocate into cells in both a perforin-dependent and independent manner. Unlike Gra-A, Gra-B shares similar substrate specificity with caspase-3 (i.e. it cleaves after aspartate residues) and induces more rapid cell death [140]. Further, Gra-B can mediate apoptosis independent of caspases, or by directly activating caspases or indirectly by activating Bid [148,149].

Apart from various other cellular substrates, Gra-B cleaves PARP-1 into 64-kD and 61-kD N-terminal fragments and C-terminal 54-kD and 42-kD fragments. Of these, the 54-kD and 42-kD PARP-1 fragments were found to be catalytically active, while the 64-kD and 61-kD fragments were inactive. This was mainly based on the presence of intact catalytic domain at the C-terminus [147]. Using activity western blots, it has been shown that the fragments 54-kD and 42-kD fragments are catalytically active. The 42-kD fragment was detected in activity western blots but not when immunoblotted with N-terminus specific antibody, indicating that it is C-terminus fragment. N-terminal analysis of the 54-kD fragment has shown that the cleavage site follows Asp^537^. It was further suggested that the appearance of 42-kD fragment is due to the secondary cleavage of 54-kD and 61-kD fragment was due to the secondary cleavage of 64-kD fragment [147].

The appearance of PARP-1 fragments with multiple molecular weights clearly distinguishes these proteases from each other, and suggests the participation of specific proteases during different phases and forms of pathology. The presence of some PARP-1 fragments, like the 50-kD and 55-kD, which can be formed by either necrotic cathepsins or by Gra-A, need to be carefully interpreted [127,145]. The PARP-1 fragments created by granzyme action are indicated in figure 1 and Additional file 1: Table-1. Based on the roles of PARP-1 in angiogenesis, whether Gra-B cleavage of PARP-1 and the resulting PARP-1 fragments have any role in modulating angiogenesis during forms of neurodegeneration is a potential opportunity for therapy.

### PARP-1 and matrix metalloproteinases

MMPs are a family of 28 zinc-dependent endopeptidases that play significant roles in angiogenesis, embryogenesis and in several pathological cardiovascular diseases like myocardial ischemia, cerebral ischemia, and atherosclerosis [150-152]. Within this family of MMPs, MMP-2 has recently been shown to be capable of cleaving PARP-1. Kwan et al. have reported that MMP-2 along with MMP-9 is present in the nuclear fraction of heart and liver, and is able to cleave PARP-1 in a concentration dependent manner. Cleavage of PARP-1 by MMP-2 produces a 66-kD and a >48-kD fragment (seen in silver stained gels and western blotting) [153] which differ in their appearance. MMP-2 produced a 66-kD PARP-1 fragment in a concentration-dependent fashion whereas there was no difference in levels of other >48-kD forms from control conditions observed in silver stained gels. Interestingly western blot analysis did not show any 66-kD PARP-1 fragments but showed an increase in >48-kD fragments in a concentration-dependent fashion. The MMP inhibitors TIMP-2 and doxycycline were able to block PARP-1 degradation to a 66-kD fragment. The reason for the appearance of a prominent ~66-kD fragment in TIMP-2 treated conditions and a faint band of the same molecular weight in doxycycline treated samples is curious. Why this fragment is specifically detected in MMP-2 inhibited samples is also unclear. Though the differences in the appearance of >48-kD and 66-kD fragments may relate to the PARP-1 antibody used (either C-terminus or N-terminus specific), the reason for the appearance of ~66-kD fragment in samples with inhibited MMP-2 (but not in untreated controls) are unclear.

MMPs actions are modulated by the cytokines and endogenous MMP inhibitors in both pro- and anti-angiogenic programs affecting extracellular and basement membrane remodeling [154]. MMP-2 is known to be a key protease involved in neovascularization, and MMP-2^-/- ^mice show defects in neovascularization. Conversely, MMP-9 may limit collagenase-induced intracerebral hemorrhage [155,156]. Moreover, conditioned medium enriched in MMP-2 and 9 (from mouse brain endothelial cells) increases the migration of neural precursor cells to sites of brain injury via ERK^1/2 ^and PI3/AKT signaling [157]. However, several CNS pathologies such as cerebral ischemia, multiple sclerosis and Devic's neuromyelitis optica are also associated with increased MMP-2 and-9 levels and MMP-9 inhibition is beneficial against cerebral ischemia [151,158]. Nicolescu et al., have recently reported that PARP-1 inhibitors can also inhibit MMPs [159], indicating that at least part of the protection afforded by PARP-1 inhibitors during stroke might be due to MMP inhibition. PARP-1 inhibition undoubtedly rescues cells from necrotic cell death, but the role(s) played by MMP inhibition in stroke clearly deserve further study.

Although acute PARP-1 and MMP inhibition may effectively attenuate ischemic stroke, the roles of MMPs in neuronal progenitor migration, and PARP-1's role in angiogenesis programs during restitution need some clarification. Importantly, whether 66-kD and >48-kD PARP-1 fragments produced by MMP proteolysis have activity or modulate PARP-1 activity is still unknown. Because PARP-1 also regulates angiogenesis, the effect of PARP-1 proteolysis by MMPs during angiogenesis may be important to investigate for cancer and chronic inflammation therapy.

## Conclusions

The molecular mechanisms involved in balancing life and death decisions controlled by PARP-1 are highly complex and incompletely understood. A delicate PARP-1 equilibrium exists within cells where any deviation, either hyper- or hypo-activity can induce or exacerbate pathology. Many related proteases including caspases, calpains, cathepsins, granzymes and MMPs also directly and indirectly mediate the effects of PARP-1 in these phenomena. Recent reports indicate that PARP-1 contributions to cell death are insult- and context-dependent. PARP-1 can also promote tissue survival by shifting the balance of cell death programs between autophagy and necrosis. PARP-1's role in driving autophagy from necrosis is distinctive from its classical role and highlights its importance in cell survival decisions. It also points out an important potential application through which modulation of PARP-1 may be applied therapeutically. Because of PARP-1's dual nature in life: death control, PARP inhibitors should be used with great caution. Furthermore, physiological and pathological roles of poly (ADP-ribosyl)ation in *parthanatos *and long term memory suggest that prolonged inhibition of PARP-1 may be ultimately deleterious. Conversely, the 24-kD N-terminal PARP-1 apoptotic fragment that attenuates necrotic cell death might eventually be developed for therapy. The appearance of specific PARP-1 fragments will not only help us understand the involvement of specific cell death proteases in particular processes, but also different types of cell death. These recent findings clearly indicate that a great deal remains be defined about PARP-1, the roles of its fragments and the specific molecular mechanisms they regulate during various forms of cell death.

## Competing interests

The authors declare that they have no competing interests.

## Authors' contributions

GVC wrote and edited the manuscript, JSA edited the manuscript and PPB edited and communicated the manuscript. All authors read and approved the final manuscript.

## Supplementary Material

Additional file 1 **Table -1**. PARP-1 signature fragments. Action of various proteases results in the generation of PARP-1 fragments with specific molecular weights that can be correlated with the action of specific proteases. PARP-1 signature fragments generated by various proteases are listed above. Click here for file
