# Supplementary material for: PARP-1 cleavage fragments: signatures of cell-death proteases in neurodegeneration
Source: Cell Commun Signal. 2010 Dec 22;8:31. doi: 10.1186/1478-811X-8-31 (PMC3022541; doi:10.1186/1478-811X-8-31)
Supplement: Additional file 1 — Table -1. PARP-1 signature fragments. Action of various proteases results in the generation of PARP-1 fragments with specific molecular weights that can be correlated with the action of specific proteases. PARP-1 signature fragments generated by various proteases are listed above. [file 1478-811X-8-31-S1.PPT]

## Slide 1
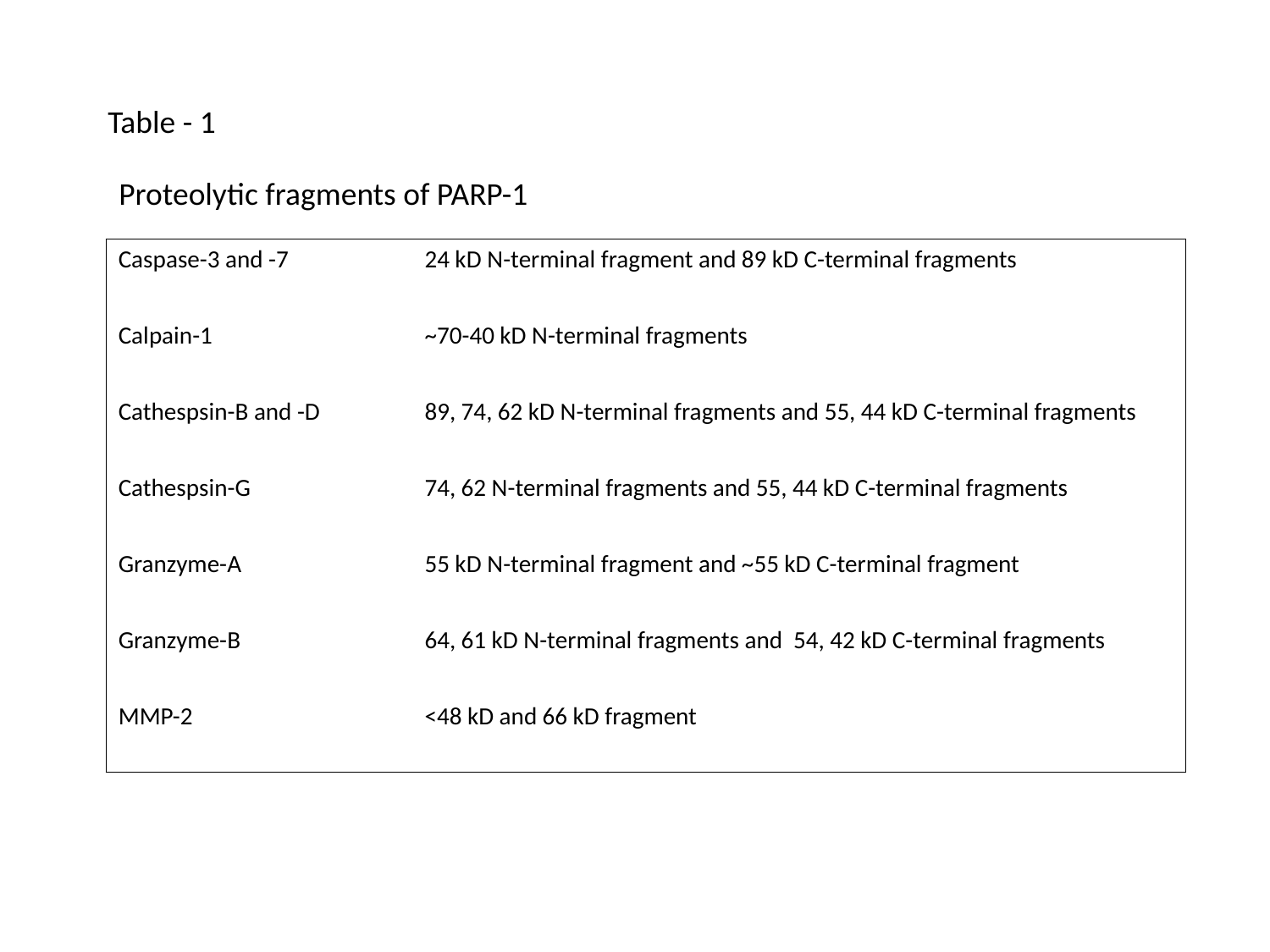

Table - 1
Proteolytic fragments of PARP-1
| Caspase-3 and -7 | 24 kD N-terminal fragment and 89 kD C-terminal fragments |
| --- | --- |
| Calpain-1 | ~70-40 kD N-terminal fragments |
| Cathespsin-B and -D | 89, 74, 62 kD N-terminal fragments and 55, 44 kD C-terminal fragments |
| Cathespsin-G | 74, 62 N-terminal fragments and 55, 44 kD C-terminal fragments |
| Granzyme-A | 55 kD N-terminal fragment and ~55 kD C-terminal fragment |
| Granzyme-B | 64, 61 kD N-terminal fragments and 54, 42 kD C-terminal fragments |
| MMP-2 | <48 kD and 66 kD fragment |
